# Assessment of postoperative circulating tumour DNA to predict early recurrence in patients with stage I–III right-sided colon cancer: prospective observational study

**DOI:** 10.1093/bjsopen/zrad146

**Published:** 2024-01-19

**Authors:** Kristin B Lygre, Rakel B Forthun, Trude Høysæter, Sigrun M Hjelle, Geir E Eide, Bjørn T Gjertsen, Frank Pfeffer, Randi Hovland

**Affiliations:** Department of Gastrointestinal Surgery, Haraldsplass Deaconess Hospital, Bergen, Norway; Department of Gastrointestinal Surgery, Haukeland University Hospital, Bergen, Norway; Department of Clinical Medicine, University of Bergen, Bergen, Norway; Department of Medicine, Haukeland University Hospital, Bergen, Norway; Section for Cancer Genomics, Haukeland University Hospital, Bergen, Norway; Section for Cancer Genomics, Haukeland University Hospital, Bergen, Norway; Department of Medical Genetics, Haukeland University Hospital, Bergen, Norway; Department of Medicine, Haukeland University Hospital, Bergen, Norway; Centre for Clinical Research, Haukeland University Hospital, Bergen, Norway; Department of Global Public Health and Primary Care, University of Bergen, Bergen, Norway; Department of Medicine, Haukeland University Hospital, Bergen, Norway; Department of Gastrointestinal Surgery, Haukeland University Hospital, Bergen, Norway; Department of Clinical Medicine, University of Bergen, Bergen, Norway; Section for Cancer Genomics, Haukeland University Hospital, Bergen, Norway; Department of Medical Genetics, Haukeland University Hospital, Bergen, Norway; Department of Biosciences, University of Bergen, Bergen, Norway

## Abstract

**Background:**

Right-sided colon cancer (RCC) differs in mutation profile and risk of recurrence compared to distal colon cancer. Circulating tumour DNA (ctDNA) present after surgery can identify patients with residual disease after curative surgery and predict risk of early recurrence.

**Methods:**

This is a prospective observational biomarker trial with exploration of ctDNA in 50 non-metastatic RCC patients for which oncological right-sided colectomy was performed. Blood samples were collected preoperatively, within 1 month post surgery, 3 months (not mandatory), 6 months and every 6 months thereafter. Plasma cell free DNA and/or tumour was investigated for cancer-related mutations by the next-generation sequencing (NGS) panel AVENIO surveillance specifically designed for ctDNA analysis. Detected mutations were quantified using digital droplet PCR (ddPCR) for follow-up. Recurrence-free survival was explored.

**Results:**

50 patients were recruited. Somatic cancer-related mutations were detected in 47/50 patients. ddPCR validated results from NGS for 27/34 (plasma) and 72/72 samples (tumour). Preoperative ctDNA was detected in 31/47 of the stage I/III patients and the majority of ctDNA positive patients showed reduction of ctDNA after surgery (27/31). ctDNA-positive patients at first postoperative sample had high recurrence risk compared to patients without measurable ctDNA (adjusted hazard ratio: 172.91; 95% c.i.: 8.70 to 3437.24; *P*: 0.001).

**Conclusion:**

ctDNA was detectable in most patients with non-metastatic RCC before surgery. Positive postoperative ctDNA was strongly associated with early recurrence. Detectable postoperative ctDNA is a prognostic factor with high (100%) positive predictive value for recurrence in this cohort of non-metastatic RCC.

**Clinical Trial Registration:**

ClinicalTrials.gov ID: NCT03776591

## Teaser

Postoperative positive ctDNA is a strong predictor for early recurrence in patients with stage I–III right-sided colon cancer.

## Introduction

Colorectal cancer remains one of the most common cancers worldwide. Incidence is 17.2/100 000 per year with annual mortality rates of 8.4/100 000^1^. In Norway, incidence of colorectal cancer is among the highest in the world^2^. Surgery is the main treatment for stages I–III. Despite improved surgical technique, pathological staging and advances in neo-adjuvant and adjuvant therapy, 30–50% of colorectal cancer patients will develop recurrence. About 25% of patients with recurrence were initially classified as stages I and II^3^.

TNM classification is part of the current risk stratification to guide recommendations regarding adjuvant chemotherapy (ACT). However, lymph node negative patients can still develop recurrence, suggesting that the TNM classification and histopathological adverse features alone are not adequate^4^. The histopathological basis of stratifying into ‘high risk’ and ‘low risk’ does not consider whether minimal residual disease (MRD) is present. Therefore, are there better methods for improving identification of patients at higher risk of recurrence? The evidence supports that circulating tumour DNA (ctDNA) can contribute to a more precise risk stratification by detecting MRD^5–8^. ctDNA is one of the most investigated and promising tumour-derived detached constituents detected in blood^9–12^. Levels of ctDNA correlate with tumour burden, and detection of postoperative ctDNA indicates presence of MRD^5–8,13^. Initial studies focused on metastatic disease and monitoring of treatment response^14^; however, there is no greater focus on non-metastatic disease and the role of liquid biopsy in predicting and detecting recurrence^9–12^.

Colorectal cancer is often presented as one disease process; however, prognosis improves the more distally the cancer is located. Right-sided colon cancer (RCC) stage III has lower survival rates and higher risk of recurrence compared to distal colorectal cancer^15–17^. Knowledge of the embryology, morphological differences, mutation profile and worse prognosis in RCC compared to distal cancers has focused research efforts on right-sided colon cancer^18,19^. The RCT ‘Open D3 Right Colectomy Compared to Laparoscopic CME for Right-Sided Colon Cancer (D3/CME)’ started recruiting in 2016 and has included liquid biopsy since 2017.

The aim of this study was to assess whether ctDNA provides additional information about prognosis beyond established risk stratification in a study population of non-metastatic RCCs.

## Methods

### Study information

This is a substudy of ‘Open D3 Right Colectomy Compared to Laparoscopic CME for Right-Sided Colon Cancer (D3/CME)’ (Clinicaltrials.gov identifier NCT03776591). The trial was conducted at Haukeland University Hospital (HUH) and Haraldsplass Deaconess Hospital (HDH), Bergen, Norway. Patients were randomized to receive either open D3 resection (HUH) or laparoscopic complete mesocolic excision (CME) with central vascular ligation (CVL) (HDH)^20^. The trial was approved by the regional committee of ethics (REK Sor-Ost, REK 2015/2396) and is in accordance with the Declaration of Helsinki^21^. This is a prospective observational biomarker trial in patients with stages I–III right-sided colon cancer and includes the first consecutive 50 patients analysed with liquid biopsies. Patients underwent surgical resection for colon cancer from September 2017 to July 2019 with clinical follow-up until 14 April 2023. Median follow-up was 4.4 years (1–5.6). Cost and practical issues limited the sample size.

Patients 18–85 years of age with non-metastatic adenocarcinoma in the right colon verified by biopsy, colonoscopy or CT were eligible for inclusion. Patients received treatment and follow-up according to national guidelines (Norwegian Directorate of Health)^22^ (*Supplementary material*).

### Sample collection

Blood samples were collected between September 2017 and January 2021, prior to and after surgery (2–7 days or 1 month, 3 months (not mandatory), 6 months, and then successively every 6 months). Plasma was separated from K_2_-EDTA blood within 1 h of blood draw by centrifuging the blood for 820*×g*, 10 min, before a second centrifugation for the supernatant at 10 000*×g*, 10 min. Purified plasma was stored at −80°C in six aliquots until further processing performed within 2 years. Biopsies from the primary tumour were collected intraoperatively by the surgeon immediately after removal of the specimen and were snap frozen as four aliquots in liquid nitrogen. Samples were stored at −150°C until time of analysis. Analysis was performed on presurgical plasma samples (*n* = 29), tumour biopsies (*n* = 45), and/or postoperative plasma samples (*n* = 34). ctDNA and tumour analysis were performed retrospectively, blinded to patient outcome.

### AVENIO ctDNA surveillance panel

Purified plasma, 3–5 ml (*n* = 62), was thawed prior to enrichment of cell-free DNA by the AVENIO ctDNA Analysis Kit (Roche) according to the protocol provided by the producers (*Supplementary material*). Sequencing libraries were prepared from 13–50 ng cell-free DNA using the AVENIO ctDNA Analysis Kit paired with the AVENIO ctDNA Surveillance Kit (Roche) as described by the manufacturers. The libraries for both ctDNA and tumour were sequenced on a NextSeq550 (Illumina) using the NextSeq 500/550 High Output v2 kit (300 cycles) (Illumina) and results were analysed using AVENIO ctDNA Analysis Software version 2.0.0 (Roche) as recommended by the suppliers, with hg38 as reference genome (*Supplementary material*). The detection threshold was 0.1% for single nucleotide variants.

### AVENIO tumour tissue surveillance panel

A Cryostat microtome was used to make 30-µm slides of fresh frozen tissue (*n* = 45). Slides from tumour were stained routinely with haematoxylin/eosin and tumour content was verified by microscopy. DNA was purified from 10 to 25 mg tissue using QIAamp DNA Mini and Blood Mini kit (Qiagen) as recommended by the manufacturer (*Supplementary material*). Sequencing libraries were prepared from 20 to 24 ng DNA harvested from fresh frozen primary biopsies, as described above, using the AVENIO Tumour Tissue Analysis Kit paired with the AVENIO Tumour Surveillance Kit (both from Roche) as recommended by the manufacturer, with minor alterations (*Supplementary material*). Detection threshold was 5% for single nucleotide variants.

### Digital droplet PCR

Cell-free DNA was harvested from 4–5 ml purified plasma (*n* = 311) using the QIAamp Circulating Nucleic Acid Kit (Qiagen) according to the protocol provided by the producer (*Supplementary material*). Digital droplet PCR (ddPCR) assays for mutations detected by the AVENIO Surveillance gene panel were purchased from Bio-Rad (*Table S1*) and ddPCR was performed as previously described^23^, with minor alterations. Briefly, all samples were run as triplicates, and results are presented as an average between replicates calculating number of mutant DNA copies per millilitre plasma and fractional abundance (FA) as mutant DNA copies/total DNA copies. Results were presented as percentage of FA. Samples with <12 000 droplets generated per parallel were excluded from further analysis. Based on validation of detection thresholds for each individual assay using positive controls, normal controls and non-template controls, samples generating a total of <3 mutation-positive droplets or having an FA < 0.1% were defined as having no detectable tumour DNA. ddPCR analysis was performed on three neoadjuvant, 46 preoperative and 262 postoperative samples (total: 311 samples, median: 7 samples per patient, range: 2–9). Twenty-five patients were assessed by two assays (53%) and 22 by one assay (47%).

### Statistical analyses

Baseline and tumour characteristics were summarized using descriptive statistics. Primary clinical endpoint examined is recurrence-free survival (RFS). Unadjusted RFS was explored using Kaplan–Meier plots^24^ and Cox regression^25^ was used for unadjusted and adjusted analyses. Results were reported as unadjusted HR or adjusted HR (aHR) with 95% c.i.s and likelihood ratio *P*. All statistical analyses were performed using SPSS version 26.0.0.1 (*Supplementary material*).

## Results

Patient and tumour characteristics are presented in *Table 1*. An overview of analysed tissue, sample time, method for analysis and results is presented in *Table 2*.

**Table 1:** Patient and tumour characteristics for *n* = 50 patients with right-sided non-metastatic colon cancer included in D3/CME-study and operated at Haraldsplass Deaconess Hospital and Haukeland University Hospital, Bergen (Norway) between September 2017 and July 2019

Patient variables	Statistic
Age (years), mean(s.d.), median (range)	69.1(10.1), 69 (39–84)
BMI (kg/m^2^), mean(s.d.), median (range)	25.6(4.3), 25.4 (17.8–38.7)
**Sex**	
Male	24
Female	26
**ASA class**	
I	5 (10)
II	40 (80)
III	5 (10)
**pTumour-stage**	
T1	1 (2)
T2	5 (10)
T3	28 (56)
T4a	15 (30)
T4b	1 (2)
**pNode-stage**	
N0	27 (54)
N1a	9 (18)
N1b	5 (10)
N1c	3 (6)
N2a	4 (8)
N2b	2 (4)
**Tumour differentiation**	
Poor	6 (12)
Moderate	43 (86)
Well	1 (2)
**Tumour morphology**	
Adenocarcinoma	49 (98)
Signet ring cell carcinoma	1 (2)
Mucinous differentiation/component	5 (10)
Tumour deposit	4 (8)
Venous invasion	12 (24)
**MSI status**	
MSS	37 (74)
MSI	11 (22)
Unknown	2 (4)
**Mutations**	
*APC*	29 (58)
*BRAF*	19 (38)
*KRAS*	25 (50)
*NRAS*	6 (12)
*TP53*	29 (58)
*PIK3CA*	12 (24)
**Neo-adjuvant chemotherapy**	
No	47 (94)
Yes	3 (6)
**Adjuvant chemotherapy**	
No	30 (60)
Yes	20 (40)
**Recurrence during follow-up**	
No	40 (80)
Yes	10 (20)
Observation time (days), mean(s.d.), median (range)	1417(354), 1448 (351–1861)
**Status (April 2023)**	
Alive without recurrence	37 (74)
Alive with recurrence	5 (10)
Dead without recurrence	3 (6)
Dead with recurrence	5 (10)

Values are *n* (%) unless otherwise stated. p, pathologic; T, tumour; N, node; MSI, microsatellite instable; MSS, microsatellite stable; *APC*, adenomatous polyposis coli gene; *BRAF*, proto-oncogene B-Raf; *KRAS*, Kirsten rat sarcoma; *NRAS*, neuroblastoma-RAS; *TP53*, tumour protein 53; *PIK3CA*, phosphatidylinositol-4,5-bisphosphate 3-kinase, catalytic subunit alpha.

**Table 2:** Analysed tissue, sampling time and method for analysis for *n* = 50 patients with right-sided non-metastatic colon cancer included in D3/CME-study and operated at Haraldsplass Deaconess Hospital and Haukeland University Hospital, Bergen (Norway) between September 2017 and July 2019

Biomarker method	Number of patients	Tumour biopsy blood (liquid biopsy)			
		Intraoperative	Prior to surgery	Postoperative*	Monitor†
AVENIO	Total	45	29	29	5
	Biomarker positive	42	22	2	1
ddPCR assay	Total	47	46	47	46
	Biomarker positive	47	23	5	8
AVENIO and/or ddPCR	Total	50	49	49	46
	Biomarker positive	49	31	5	8

AVENIO: AVENIO ctDNA targeted kit; ctDNA: circulating tumor DNA; ddPCR: digital droplet polymerase chain reaction. *Postoperative sample between 2 and 83 days after operation. Forty/50 patients were sampled within the first postoperative week. For the 10 patients with delayed sampling, four received chemotherapy and were sampled after initiation of treatment. Mean cycles before sampling: 1.75. Mean time from last treatment: 17.5 days. †Monitor includes all time points after operation excluding the first postoperative sampling, sampling at 3–38 months after operation.

### Biomarker detection by next-generation sequencing

Cancer-specific mutations were found in 49/50 patients (98%). Of these, next-generation sequencing (NGS) of the tumour biopsy was possible for 45 patients. Mutation profiles were provided by NGS of plasma for the other four patients (*Table 2*).

The patient with no cancer-specific mutations detected underwent colonic resection after incomplete removal of a malignant polyp (T1sm2/3). There was no remaining tumour at the time of formal resection and the first sampling was after polypectomy. Forty-seven of 50 patients (94%) had one to two mutations eligible for monitoring with commercially available ddPCR assays.

The genes most currently mutated were *APC* (29 patients, 58%), *TP53* (29 patients, 58%), *KRAS* (25 patients, 50%), *BRAF* (19 patients, 38%), *PIK3CA* (12 patients, 24%) and *NRAS* (6 patients, 12%) (*Table S1*).

### ctDNA monitoring by ddPCR

ddPCR is more cost-effective for monitoring ctDNA than NGS, with high sensitivity and high negative predictive value given a known mutation profile^26^. To evaluate the utility of ddPCR for MRD monitoring in stage I–III patients, plasma and tumour samples were analysed by selected ddPCR markers based on mutations identified by NGS analysis in tumour and/or plasma (*Table S2*).

Tumour samples from patients with mutations eligible for monitoring by commercially available ddPCR assays (*n* = 47) were tested by 72 ddPCR analyses in total using 21 different assays. All mutations found by NGS in either plasma or tumour were confirmed present by ddPCR in the tumour (*Table S2*), showing mutational concordance between pretreatment samples and tissue. ddPCR was possible for 26 of the 29 preoperative plasma samples initially analysed by NGS. Samples were analysed by 34 ddPCR analyses in total using 13 different assays. NGS and ddPCR returned concurring results for 27/34 analyses (79% concordance). ctDNA was not detected by ddPCR in plasma samples found negative by NGS, despite tumour biopsies being mutation positive.

### Monitoring ctDNA in pre- and postoperative cell-free plasma DNA

Median (range) preoperative plasma cell-free DNA concentration was 11.3 (4.3–63.5) ng/ml plasma (*n* = 47). Postoperative cell-free DNA concentration was 38.95 (12.1–145.6) ng/ml plasma for patients sampled 2–7 days post surgery (*n* = 38), and 7.9 (6.7–18.9) ng/ml plasma for patients sampled 1–2 months post surgery (*n* = 9).

Preoperatively, 31/47 (66%) of stage I–III patients were ctDNA-positive using NGS and/or ddPCR (*Table 2*). Monitoring by ddPCR during follow-up demonstrated that 42 patients were negative for ctDNA, whereas five patients were positive in the first postoperative sample. During surveillance, 38 patients remained negative, eight patients were positive for ctDNA or became positive (four not analysed). Ten patients had recurrent cancer during surveillance (*Table 3*). Five of 10 patients with recurrence were positive for ctDNA in their first postoperative sample, and an additional three patients became positive during monitoring (34, 22 and 9 months after surgery), two of whom had positive ctDNA prior to radiological confirmation, and one after. One patient that developed lung metastasis and one with metastasis to the liver and peritoneum/retroperitoneum remained negative in analysed postoperative samples^27^. Patients with recurrence and positive ctDNA in the first postoperative sample had a mean RFS of 0.5 years (180 days). Patients with recurrence and negative ctDNA in the first postoperative sample had a mean RFS of 2.3 years (831 days).

**Table 3:** Characteristics of 10 patients (a–j) with recurrent cancer after oncologic resection of right-sided colon cancer. Patients were operated from September 2017 until July 2019 and with follow-up until 31 October 2022

Variable	Patient a	Patient b	Patient c	Patient d	Patient e	Patient f	Patient g	Patient h	Patient i	Patient j
Neo-ACT (cycles)	No	No	No	No	No	No	No	Yes (3)	No	No
T-stage	T4a	T4a	T3	T3	T3	T3	T4a	T3	T3	T4a
N-stage	N0	N2b	N1a	N0	N2a	N2a	N1c	N2a	N1a	N0
Differentiation	Low	Middle	Middle	Middle	Low	Middle	Middle	Middle	Middle	High
Morphology	AC	AC	AC	AC	SRCC	AC	AC	AC	AC	AC
Mucinous diff	Yes	No	No	No	Yes	No	No	No	No	No
Tumour deposit	No	No	Yes	No	No	No	Yes	No	No	No
Venous invasion	No	No	Yes	No	Yes	Yes	Yes	Yes	No	Yes
MSI-status	MSS	MSS	MSS	MSS	MSS	MSS	MSS	MSS	MSS	MSS
ddPCR monitoring marker	*BRAF*	*BRAF*/*TP53*	*BRAF*	*KRAS*/PIK3CA*	*BRAF*/*TP53*	*APC*/*KRAS*	*APC*/*NRAS*	*KRAS*/*APC*	*TP53*	*TP53*
Source NGS	T/plasma	T/plasma	T/plasma	T	T/plasma	Plasma	T	T	T	T
ACT (cycles)	No	Yes (3)	Yes (1)	No	No	Yes (1)	No	Yes (4)	Yes (12)	Yes (12)
ctDNA postop† (days‡)	Neg§ (1032)	Pos (3)	Pos (4)	Neg (−)	Neg§ (685)	Neg§ (257)	Pos (107)	Pos (3)	Pos (3)	Neg (−)¶
RFS in days#	1647	310	41	761	235	448	105	259	186	1063
Site of recurrence	Per	Liver/per	Liver	Lung	Retro	Liver/lung	Liver/retro	Lung	Liver	Liver/per/retro
Status 14 April 2023	Alive	Dead	Dead	Alive	Dead	Dead	Dead	Alive	Alive	Alive

ACT, adjuvant chemotherapy; T, tumour; N, node; AC, adenocarcinoma; SRCC, signet ring cell carcinoma; MSI, micro satellite instable; MSS, microsatellite stable; ddPCR, digital droplet PCR; *BRAF*, proto-oncogene B-Raf; *TP53*, tumour protein 53; *KRAS*, Kirsten rat sarcoma; *PIK3CA*, phosphatidylinositol-4,5-bisphosphate 3-kinase, catalytic subunit alpha; *APC*, adenomatous polyposis coli gene; *NRAS*, neuroblastoma-RAS; NGS, next-generation sequencing; ctDNA, circulating tumour DNA; Neg, negative; Pos, positive; RFS, recurrence-free survival; per, peritoneum; retro, retroperitoneal. *A monitorable *PIK3CA* hotspot mutation was detected. However, a *KRAS* mutation with higher VAF was selected for monitoring. †In the first postoperative sample. ‡Days from operation to positive ctDNA. §Turned positive during monitoring. ¶Last analysed sample was more than one year before detected recurrence. #Days from operation to radiologically verified recurrence.

### Clinical validity of ctDNA analysis

**Fig. 1:**
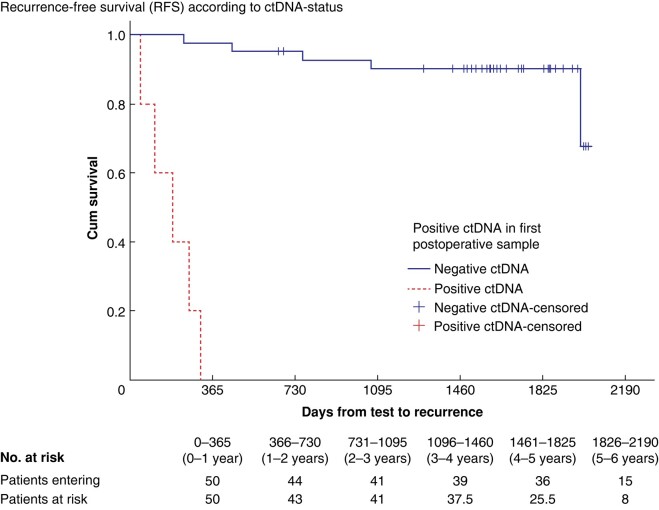
Kaplan–Meier plot for days from postoperative sampling (liquid biopsy) until postoperative recurrence in 50 patients operated for right-sided non-metastatic colon cancer in the D3/CME-study at Haraldsplass Deaconess Hospital or Haukeland University Hospital, Bergen (Norway) between September 2017 and July 2019 according to postoperative ctDNA status (*P* = 0.001)

No correlation was found between preoperative positive ctDNA and recurrence rate. Positive postoperative ctDNA was associated with recurrence (aHR: 172.91; 95% c.i.: 8.70 to 3437.24; *P*: 0.001). None of the traditional histopathological variables demonstrated association with recurrence (Cox regression). Morphology and venous invasion were the only negative histopathological prognostic factors in the unadjusted analysis. They were not significant in the adjusted model (*Table 4*) and are not included in traditional risk stratification. Kaplan–Meier plots for RFS and postoperative ctDNA status are presented in *Fig. 1*.

**Table 4:** Results from Cox regression analyses of the risk of recurrence based on traditional histopathological characteristics and presence of pre- and postoperative ctDNA in 50 patients with right-sided non-metastatic colon cancer included in D3/CME-study and operated between September 2017 and July 2019

Variables	Unadjusted models				Adjusted model		
	*n*	HR	95% c.i.	*P*	aHR	95% c.i.	*P*
**ctDNA positive**							
Preoperative	46	1.28	0.34,4.76	0.714	n.i.	n.i.	n.i.
Postoperative	47	91.37	10.22,817.26	<0.001	172.91	8.70,3437.24	0.001
T4 *versus* T1-3	50	1.45	0.41,5.16	0.564	0.66	0.12,3.68	0.637
N-positive*	50	3.85	0.99,15.01	0.052	0.95	0.12,7.25	0.959
T differentiation†	50	0.72	0.14,3.71	0.698	0.40	0.05,3.38	0.398
Morphology‡	50	15.83	1.65,152.17	0.017	n.i.	n.i.	n.i.
Mucinous diff	50	2.00	0.42,9.65	0.386	6.26	0.70,55.89	0.100
Tumour deposit	50	3.62	0.71,18.35	0.121	4.62	0.47,45.86	0.192
Venous invasion	50	5.93	1.67,21.09	0.006	4.13	0.93,18.29	0.062
MSI/MSS§	48	n.i.	n.i.	n.i.	n.i	n.i.	n.i.

*N-stage: N-positive *versus* N-negative. †Middle/well *versus* poor. ‡Adenocarcinoma *versus* Signet ring cell carcinoma. Only one patient with Signet ring cell carcinoma: this patient had recurrence. §Not possible to analyse because all patients with recurrence were MSS. aHR, adjusted HR; ctDNA, circulating tumour DNA; T, tumour; N, node; MSI/MSS, microsatellite instable/stable; n.i., not included in the model.

## Discussion

The aim of this substudy was to investigate whether ctDNA provided additional information about prognosis beyond established risk stratification, and to explore best timing and source of primary gene mapping. RCC was chosen based on adverse prognosis and its distinct biology compared to more distal colorectal cancers. The study confirmed that both tumour and plasma are good sources for primary gene mapping. ctDNA was detectable preoperatively in 66% of stage I–III RCC^28^. Furthermore, mutations detected by NGS could be confirmed and monitored by ddPCR. The major finding was that the presence of postoperative ctDNA is a strong predictor for early recurrence.

Mutated key oncogenes and tumour suppression genes were comparable with rates from The Cancer Genome Atlas Dataset. There was a higher proportion of *TP53* (58% *versus* 34.8%) and *BRAF* (38% *versus* 24.2%) mutations than other materials with RCC (transverse excluded). The presence of *APC* (58% *versus* 63.6%), *KRAS* (50% *versus* 45.5%), *PIK3CA* (24% *versus* 27.3%) and *NRAS* (12% *versus* 7.6%) was comparable^29^.

ctDNA constitutes a small portion of total cell-free DNA, in some studies less than 1%^30^. The half-life of cell-free DNA is short (minutes to hours). High levels of cell-free DNA (median 38.95 ng/ml) early postoperatively (2–7 days), due to surgical trauma, dilute the ctDNA concentration and make it difficult to detect cancer specific mutations. The dilution effect is less relevant for patients with tumours that shed high levels of ctDNA, but for patients with low tumour burden and less ctDNA, the dilution effect can result in undetectable ctDNA in the early postoperative period. Negative postoperative ctDNA should be interpreted with caution as it may be related to detection threshold. One month postoperatively, levels of cell-free DNA dropped significantly (median 7.9 ng/ml). As ACT should be initiated within 6 weeks, 4 weeks after operation is a good time point for postoperative sampling^31^.

NGS and ddPCR are complementary methods for monitoring ctDNA. With a unique molecular identifier and digital error suppression, NGS is highly sensitive and gives a broad mapping of the genetic profile. However, it is labour-intensive and expensive^32^. ddPCR is sensitive, robust and cost-effective for detection of selected mutations, but holds potential for missing relevant mutations by selection of a suboptimal surveillance marker. To identify MRD with targeted analyses like RT-PCR/ddPCR/BEAMing, the selected mutation must be present in all cancer cells. It is known that intra-tumour mutational heterogeneity can be present^33,34^. Many ctDNA studies choose a tumour-agnostic approach with selection of surveillance mutations without knowledge of the actual mutation profile^30^. Using this strategy, a negative result by ddPCR is not synonymous with negative ctDNA or no MRD present. It only confirms that the selected mutation is not present. In this trial, we chose an approach with a broad-coverage NGS assay for initial mutation profiling for plasma, tumour or both. This approach increased the probability of detection of relevant mutations and allowed monitoring of eight patients (17% of the patient cohort) lacking classical codon 600 *BRAF* and codon 12/13/61 *KRAS* mutations. Without *a priori* knowledge of tumour genotype, they would not be included. Monitoring of ctDNA was performed by ddPCR, and criteria for selection of surveillance markers were that mutations were detected by NGS and confirmed by ddPCR (requires commercially available assays). Concordance between NGS and ddPCR was high with 100% confirmation of NGS with ddPCR for tumour and 79% for plasma. Selection bias was reduced with a broad NGS-based approach, and the high concordance and cost-effectiveness of ddPCR makes it possible to implement in routine diagnostics, especially for surveillance.

Initial genomic profiling can be conducted on plasma or tumour tissue. Plasma has been shown to be a good option for patients receiving neo-adjuvant treatment before surgery^35^, and is promising for cancer patients when tumour biopsies are not available. Theoretically, plasma will reflect intra-tumoural heterogeneity better than single tumour biopsies^36–39^. However, there was little discrepancy between variants selected for monitoring by NGS in tumour and plasma (one mutation detected in plasma and not tumour). There was little additional gain in capturing tumour heterogeneity by performing NGS on plasma rather than tumour. Both plasma and tumour could be reference material for detecting markers for monitoring, even in cases where only one tumour biopsy was analysed. In addition to intra-tumoural heterogeneity, there is a risk of altered mutation profile due to clonal selection during treatment and surveillance^40,41^. To increase the likelihood of capturing relevant changes, we followed two variants when possible. Due to the limited number of patients and sampling period in this trial, evaluation of the role of ctDNA as a diagnostic tool for early detection of recurrence was restricted and we cannot evaluate the predictive precision of ctDNA during surveillance. Analysis of the remaining study population, with complete surveillance of 5 years, may clarify this.

As surgery is a curative treatment for limited disease, theoretically, successful surgery would lead to undetectable postoperative ctDNA. Exploration of MRD after surgery is not included in traditional risk assessment, and tools for surveillance are limited to less-sensitive diagnostic tools such as carcinoembryonic antigen (CEA) measurement and CT imaging. CEA does not detect recurrence at an early stage^35,42,43^, whereas CT scanning has a threshold of 5–10 mm for detection of lesions and often yields unspecific findings^44–46^. In accordance with previous studies^6,7,47,48^, this trial confirms that ctDNA is a marker for MRD. Early postoperative ctDNA positivity was associated with risk of recurrence, whereas traditional risk stratification variables were non-significant. Improvements can be made to the current method for selection of patients for ACT in colon cancer. ctDNA holds potential to guide a tailored adjuvant treatment decision. The clinical breakthrough would be if ctDNA status could identify the stage III patients who will not benefit from ACT and select high-risk stage II patients who might benefit. The potential for downscaling of ACT or possibly skipping adjuvant treatment is currently being further explored in clinical intervention trials with ctDNA-guided management^4,49–54^. Only 5 of 10 patients with recurrence were positive for ctDNA in their first postoperative sample. Recurrence risk was low in the negative group (11% negative *versus* 100% positive). Additional information from ctDNA status compared to established risk stratification is difficult to interpret, especially for ctDNA-negative patients. Postoperative ctDNA status alone cannot yet guide treatment decisions, but can supplement traditional risk stratification. ctDNA is a reliable predictor for early recurrence by detecting MRD.

In summary, ctDNA was detectable prior to surgery for most patients with stages I–III RCC, but its presence was not predictive for negative outcome. Postoperative (2–83 days) positive ctDNA was a marker for MRD and a predictor for early recurrence. The clinical utility remains to be proven in clinical intervention trials.

## Supplementary Material

zrad146_Supplementary_Data

## Data Availability

The authors confirm that the data supporting the findings of this study are available within the article and its Supplementary materials. Raw data are available from the corresponding author upon reasonable request.

## References

[1] Bray F, Ferlay J, Soerjomataram I, Siegel RL, Torre LA, Jemal A. Global cancer statistics 2018: GLOBOCAN estimates of incidence and mortality worldwide for 36 cancers in 185 countries. CA Cancer J Clin 2018;68:394–424 30207593 10.3322/caac.21492

[2] Kreftregisteret . Cancer in Norway 2018 Cancer incidence, mortality, survival and prevalence in Norway 2018 (kreftregisteret.no).

[3] Young PE, Womeldorph CM, Johnson EK, Maykel JA, Brucher B, Stojadinovic A et al Early detection of colorectal cancer recurrence in patients undergoing surgery with curative intent: current status and challenges. J Cancer 2014;5:262–271 24790654 10.7150/jca.7988 PMC3982039

[4] Tie J, Cohen JD, Lahouel K, Lo SN, Wang Y, Kosmider S et al Circulating tumor DNA analysis guiding adjuvant therapy in stage II colon cancer. N Engl J Med 2022;386:2261–2272 35657320 10.1056/NEJMoa2200075 PMC9701133

[5] Bach S, Sluiter NR, Beagan JJ, Mekke JM, Ket JCF, van Grieken NCT et al Circulating tumor DNA analysis: clinical implications for colorectal cancer patients. A systematic review. JNCI Cancer Spectr 2019;3:pkz042 32328554 10.1093/jncics/pkz042 PMC7050033

[6] Tie J, Wang Y, Tomasetti C, Li L, Springer S, Kinde I et al Circulating tumor DNA analysis detects minimal residual disease and predicts recurrence in patients with stage II colon cancer. Sci Transl Med 2016;8:346ra92 10.1126/scitranslmed.aaf6219 PMC5346159 27384348

[7] Tie J, Cohen JD, Wang Y, Christie M, Simons K, Lee M et al Circulating tumor DNA analyses as markers of recurrence risk and benefit of adjuvant therapy for stage III colon cancer. JAMA Oncol 2019;5:1710–1717 31621801 10.1001/jamaoncol.2019.3616 PMC6802034

[8] Diehn M, Alizadeh AA, Adams H-P, Lee JJ, Klassen S, Palma JF et al Early prediction of clinical outcomes in resected stage II and III colorectal cancer (CRC) through deep sequencing of circulating tumor DNA (ctDNA). J Clin Oncol 2017;35:359128892431

[9] Wan JCM, Massie C, Garcia-Corbacho J, Mouliere F, Brenton JD, Caldas C et al Liquid biopsies come of age: towards implementation of circulating tumour DNA. Nat Rev Cancer 2017;17:223–238 28233803 10.1038/nrc.2017.7

[10] Ignatiadis M, Lee M, Jeffrey SS. Circulating tumor cells and circulating tumor DNA: challenges and opportunities on the path to clinical utility. Clin Cancer Res 2015;21:4786–4800 26527805 10.1158/1078-0432.CCR-14-1190

[11] Nordgård O, Tjensvoll K, Gilje B, Søreide K. Circulating tumour cells and DNA as liquid biopsies in gastrointestinal cancer. Br J Surg 2018;105:e110–e120 29341153 10.1002/bjs.10782

[12] Rasmussen SL, Krarup HB, Sunesen KG, Pedersen IS, Madsen PH, Thorlacius-Ussing O. Hypermethylated DNA as a biomarker for colorectal cancer: a systematic review. Colorectal Dis 2016;18:549–561 26998585 10.1111/codi.13336

[13] Schøler LV, Reinert T, Ørntoft M-BW, Kassentoft CG, Árnadóttir SS, Vang S et al Clinical implications of monitoring circulating tumor DNA in patients with colorectal cancer. Clin Cancer Res 2017;23:5437–5445 28600478 10.1158/1078-0432.CCR-17-0510

[14] de Figueiredo Barros BD, Kupper BEC, Aguiar Junior S, de Mello CAL, Begnami MD, Chojniak R et al Mutation detection in tumor-derived cell free DNA anticipates progression in a patient with metastatic colorectal cancer. Front Oncol 2018;8:306 30148116 10.3389/fonc.2018.00306 PMC6095987

[15] Wang CB, Shahjehan F, Merchea A, Li Z, Bekaii-Saab TS, Grothey A et al Impact of tumor location and variables associated with overall survival in patients with colorectal cancer: a Mayo Clinic Colon and Rectal Cancer Registry study. Front Oncol 2019;9:76 30838175 10.3389/fonc.2019.00076 PMC6389639

[16] Qin Q, Yang L, Sun YK, Ying JM, Song Y, Zhang W et al Comparison of 627 patients with right- and left-sided colon cancer in China: differences in clinicopathology, recurrence, and survival. Chronic Dis Transl Med 2017;3:51–59 29063056 10.1016/j.cdtm.2017.02.004 PMC5627696

[17] Arnold D, Lueza B, Douillard JY, Peeters M, Lenz HJ, Venook A et al Prognostic and predictive value of primary tumour side in patients with *RAS* wild-type metastatic colorectal cancer treated with chemotherapy and EGFR directed antibodies in six randomized trials. Ann Oncol 2017;28:1713–1729 28407110 10.1093/annonc/mdx175 PMC6246616

[18] Song Y, Wang L, Ran W, Li G, Xiao Y, Wang X et al Effect of tumor location on clinicopathological and molecular markers in colorectal cancer in eastern China patients: an analysis of 2,356 cases. Front Genet 2020;11:96 32161617 10.3389/fgene.2020.00096 PMC7052354

[19] Mukund K, Syulyukina N, Ramamoorthy S Subramaniam S. Right and left-sided colon cancers—specificity of molecular mechanisms in tumorigenesis and progression. BMC Cancer 2020;20:317 32293332 10.1186/s12885-020-06784-7 PMC7161305

[20] Lygre KB, Eide GE, Forsmo HM, Dicko A, Storli KE, Pfeffer F. Complications after open and laparoscopic right-sided colectomy with central lymphadenectomy for colon cancer: randomized controlled trial. BJS Open 2023;7:zrad074 37643373 10.1093/bjsopen/zrad074 PMC10465081

[21] World Medical Association . World Medical Association Declaration of Helsinki: ethical principles for medical research involving human subjects. JAMA 2013;310:2191–2194 24141714 10.1001/jama.2013.281053

[22] Helsedirektoratet . Nasjonalt handlingsprogram med retningslinjer for diagnostikk, behandling og oppfølging av kreft i tykktarm og endetarm: 2017. http://www.helsebiblioteket.no/retningslinjer/kreft-i-tykktarm-og-endetarm/8-tykktarmskreft-u.metastaser/8.6-adjuvant-kjemoterapi.

[23] Forthun RB, Hovland R, Schuster C, Puntervoll H, Brodal HP, Namløs HM et al ctDNA detected by ddPCR reveals changes in tumour load in metastatic malignant melanoma treated with bevacizumab. Sci Rep 2019;9:17471 31767937 10.1038/s41598-019-53917-5 PMC6877652

[24] Kaplan EL, Meier P. Nonparametric estimation from incomplete observations. J Am Stat Assoc 1958;53:457–481

[25] Cox DR . Regression models and life-tables. J R Stat Soc Ser B Methodol 1972;34:187–202

[26] Tsao SC-H, Weiss J, Hudson C, Christophi C, Cebon J, Behren A et al Monitoring response to therapy in melanoma by quantifying circulating tumour DNA with droplet digital PCR for *BRAF* and *NRAS* mutations. Sci Rep 2015;5:11198 26095797 10.1038/srep11198 PMC4476039

[27] Hamfjord J, Guren TK, Glimelius B, Sorbye H, Pfeiffer P, Dajani O et al Clinicopathological factors associated with tumour-specific mutation detection in plasma of patients with *RAS*-mutated or *BRAF*-mutated metastatic colorectal cancer. Int J Cancer 2021;149:1385–1397 33961700 10.1002/ijc.33672

[28] Hofste LSM, Geerlings MJ, von Rhein D, Rütten H, Westenberg AH, Weiss MM et al Circulating tumor DNA detection after neoadjuvant treatment and surgery predicts recurrence in patients with early-stage and locally advanced rectal cancer. Eur J Surg Oncol 2023;49:1283–1290 36740555 10.1016/j.ejso.2023.01.026

[29] Lee MS, Menter DG, Kopetz S. Right *versus* left colon cancer biology: integrating the consensus molecular subtypes. J Natl Compr Canc Netw 2017;15:411–419 28275039 10.6004/jnccn.2017.0038

[30] Diehl F, Li M, Dressman D, He Y, Shen D, Szabo S et al Detection and quantification of mutations in the plasma of patients with colorectal tumors. Proc Natl Acad Sci USA 2005;102:16368–16373 16258065 10.1073/pnas.0507904102 PMC1283450

[31] Cohen SA, Kasi PM, Aushev VN, Hanna DL, Botta GP, Sharif S et al Kinetics of postoperative circulating cell-free DNA and impact on minimal residual disease detection rates in patients with resected stage I–III colorectal cancer. J Clin Oncol 2023;41:5

[32] Newman AM, Lovejoy AF, Klass DM, Kurtz DM, Chabon JJ, Scherer F et al Integrated digital error suppression for improved detection of circulating tumor DNA. Nat Biotechnol 2016;34:547–555 27018799 10.1038/nbt.3520 PMC4907374

[33] Morris LGT, Riaz N, Desrichard A, Şenbabaoğlu Y, Hakimi AA, Makarov V et al Pan-cancer analysis of intratumor heterogeneity as a prognostic determinant of survival. Oncotarget 2016;7:10051–10063 26840267 10.18632/oncotarget.7067 PMC4891103

[34] McGranahan N, Swanton C. Clonal heterogeneity and tumor evolution: past, present, and the future. Cell 2017;168:613–628 28187284 10.1016/j.cell.2017.01.018

[35] Parikh AR, Van Seventer EE, Siravegna G, Hartwig AV, Jaimovich A, He Y et al Minimal residual disease detection using a plasma-only circulating tumor DNA assay in patients with colorectal cancer. Clin Cancer Res 2021;27:5586–5594 33926918 10.1158/1078-0432.CCR-21-0410 PMC8530842

[36] Navin N, Krasnitz A, Rodgers L, Cook K, Meth J, Kendall J et al Inferring tumor progression from genomic heterogeneity. Genome Res 2010;20:68–80 19903760 10.1101/gr.099622.109 PMC2798832

[37] Gerlinger M, Rowan AJ, Horswell S, Math M, Larkin J, Endesfelder D et al Intratumor heterogeneity and branched evolution revealed by multiregion sequencing. N Engl J Med 2012;366:883–892 22397650 10.1056/NEJMoa1113205 PMC4878653

[38] Swanton C . Intratumor heterogeneity: evolution through space and time. Cancer Res 2012;72:4875–4882 23002210 10.1158/0008-5472.CAN-12-2217 PMC3712191

[39] Rajput A, Bocklage T, Greenbaum A, Lee J-H, Ness SA. Mutant-allele tumor heterogeneity scores correlate with risk of metastases in colon cancer. Clin Colorectal Cancer 2017;16:e165–e170 28073683 10.1016/j.clcc.2016.11.004 PMC5441963

[40] Diaz LA Jr, Williams RT, Wu J, Kinde I, Hecht JR, Berlin J et al The molecular evolution of acquired resistance to targeted EGFR blockade in colorectal cancers. Nature 2012;486:537–540 22722843 10.1038/nature11219 PMC3436069

[41] Li C, Xu J, Wang X, Zhang C, Yu Z, Liu J et al Whole exome and transcriptome sequencing reveal clonal evolution and exhibit immune-related features in metastatic colorectal tumors. Cell Death Discov 2021;7:222 34453042 10.1038/s41420-021-00607-9 PMC8397721

[42] Sørensen CG, Karlsson WK, Pommergaard H-C, Burcharth J, Rosenberg J. The diagnostic accuracy of carcinoembryonic antigen to detect colorectal cancer recurrence—a systematic review. Int J Surg 2016;25:134–144 26700203 10.1016/j.ijsu.2015.11.065

[43] Kotani D, Oki E, Nakamura Y, Yukami H, Mishima S, Bando H et al Molecular residual disease and efficacy of adjuvant chemotherapy in patients with colorectal cancer. Nat Med 2023;29:127–134 36646802 10.1038/s41591-022-02115-4 PMC9873552

[44] Ko Y, Kim J, Park JK-H, Kim H, Jai Young C, Sung-Bum K et al Limited detection of small (≤10 mm) colorectal liver metastasis at preoperative CT in patients undergoing liver resection. PLoS One 2017;12: e0189797 29244853 10.1371/journal.pone.0189797 PMC5731738

[45] Regge D, Campanella D, Anselmetti GC, Cirillo S, Gallo TM, Muratore A et al Diagnostic accuracy of portal-phase CT and MRI with mangafodipir trisodium in detecting liver metastases from colorectal carcinoma. Clin Radiol 2006;61:338–347 16546464 10.1016/j.crad.2005.12.010

[46] Chung C-C, Hsieh C-C, Lee H-C, Wu M-H, Huang M-H, Hsu W-H et al Accuracy of helical computed tomography in the detection of pulmonary colorectal metastases. J Thorac Cardiovasc Surg 2011;141:1207–1212 21130470 10.1016/j.jtcvs.2010.09.052

[47] Reinert T, Henriksen TV, Christensen E, Sharma S, Salari R, Sethi H et al Analysis of plasma cell-free DNA by ultradeep sequencing in patients with stages I to III colorectal cancer. JAMA Oncol 2019;5:1124–1131 31070691 10.1001/jamaoncol.2019.0528 PMC6512280

[48] Tie J, Cohen JD, Wang Y, Lu L, Christie M, Simons K et al Serial circulating tumour DNA analysis during multimodality treatment of locally advanced rectal cancer: a prospective biomarker study. Gut 2019;68:663–671 29420226 10.1136/gutjnl-2017-315852 PMC6265124

[49] Taïeb J, Benhaim L, Laurent Puig P, Le Malicot K, Emile JF, Geillon F et al Decision for adjuvant treatment in stage II colon cancer based on circulating tumor DNA: the CIRCULATE-PRODIGE 70 trial. Dig Liver Dis 2020;52:730–733 32482534 10.1016/j.dld.2020.04.010

[50] Folprecht G, Reinacher-Schick A, Weitz J, Lugnier C, Kraeft A-L, Wisser S et al The CIRCULATE trial: circulating tumor DNA based decision for adjuvant treatment in colon cancer stage II evaluation (AIO-KRK-0217). Clin Colorectal Cancer 2022;21:170–174 34772609 10.1016/j.clcc.2021.09.005

[51] Taniguchi H, Nakamura Y, Kotani D, Yukami H, Mishima S, Sawada K et al CIRCULATE-Japan: circulating tumor DNA–guided adaptive platform trials to refine adjuvant therapy for colorectal cancer. Cancer Sci 2021;112:2915–2920 33931919 10.1111/cas.14926 PMC8253296

[52] Morris VK, Yothers G, Kopetz S, Jacobs SA, Lucas PC, Iqbal A et al Phase II/III study of circulating tumor DNA as a predictive biomarker in adjuvant chemotherapy in patients with stage II colon cancer: nRG-GI005 (COBRA). J Clin Oncol 2021;39:TPS3622-TPS

[53] Schraa SJ, van Rooijen KL, van der Kruijssen DEW, Rubio Alarcón C, Phallen J, Sausen M et al Circulating tumor DNA guided adjuvant chemotherapy in stage II colon cancer (MEDOCC-CrEATE): study protocol for a trial within a cohort study. BMC Cancer 2020;20:790 32819390 10.1186/s12885-020-07252-y PMC7441668

[54] Anandappa G, Starling N, Peckitt C, Bryant A, Begum R, Carter P et al TRACC: tracking mutations in cell-free DNA to predict relapse in early colorectal cancer—a randomized study of circulating tumour DNA (ctDNA) guided adjuvant chemotherapy *versus* standard of care chemotherapy after curative surgery in patients with high risk stage II or stage III colorectal cancer (CRC). J Clin Oncol 2020;38:TPS4120-TPS

