# Supplementary material for: Assessment of postoperative circulating tumour DNA to predict early recurrence in patients with stage I–III right-sided colon cancer: prospective observational study
Source: BJS Open. 2024 Jan 19;8(1):zrad146. doi: 10.1093/bjsopen/zrad146 (PMC10799327; doi:10.1093/bjsopen/zrad146)
Supplement: zrad146_Supplementary_Data [file zrad146_supplementary_data.docx]

**Assessment of post-operative circulating tumour DNA to predict early recurrence in patients with stage I-III right-sided colon cancer: Prospective observational study**

Kristin B Lygre^1, 2, 3*^; Rakel B Forthun^4, 5^*; Trude Høysæter^5, 6^; Sigrun M Hjelle^4^; Geir E Eide ^7, 8^; Bjørn T Gjertsen^4^; Frank Pfeffer^2, 3^; Randi Hovland^5,6 9#^

^1^ Department of Gastrointestinal Surgery, Haraldsplass Deaconess Hospital, Bergen, Norway;

^2^ Department of Gastrointestinal Surgery, Haukeland University Hospital, Bergen, Norway;

^3^ Department of Clinical Medicine, University of Bergen, Bergen, Norway;

^4^ Department of Medicine, Haukeland University Hospital, Bergen, Norway;

^5^ Section for Cancer Genomics, Haukeland University Hospital, Bergen, Norway;

^6^ Department of Medical Genetics, Haukeland University Hospital, Bergen, Norway;

^7^ Centre for Clinical Research, Haukeland University Hospital, Berge, Norway;

^8^Department of Global Public Health and Primary Care, University of Bergen, Bergen, Norway;

^9^ Department of Biosciences, University of Bergen, Bergen, Norway

*These authors contributed equally to this paper

**Corresponding author.** Randi Hovland, Section for Cancer Genomics, Haukeland University Hospital, Bergen, Norway, TEL: +47 55975322 e-mail: [Randi.Hovland@helse-bergen.no](mailto:Randi.Hovland@helse-bergen.no), **ORCID ID** 0000-0001-8543-2213

**Supplementary Materials - Index**

| **Supplementary Methods** |  |
| --- | --- |
| Study information –patient cohort | *pag. 3* |
| Cell free DNA purification for AVENIO NGS-panel | *pag. 3* |
| Avenio ctDNA Surveillance Panel Library preparation | *pag. 3* |
| Tumour biopsy DNA purification for AVENIO Surveillance tissue kit | *pag. 4* |
| Avenio Tumour Tissue Surveillance Panel Library preparation | *pag. 4* |
| Bioinformatics | *pag. 4* |
| Digital droplet PCR | *pag.5* |
| Microsatellite instability (MSI) analysis | *pag.5* |
| Statistical analysis | *pag.5* |
|  |  |
|  |  |
| **Supplementary Figures and Tables** |  |
| Supplemetary Table S1 | *pag. 6* |
| Supplementary Table S2 | *pag. 13* |
| Supplementary Table S3 | *pag. 16* |
| Supplementary Figure 1 | *pag. 20* |

**Supplementary Methods**

*Study information –patient cohort*

Definition of right-sided colon cancer is tumours evolving from the embryological mid-gut (caecum, ascending colon and the proximal 2/3 of transverse colon). Patients were medically cleared for general anaesthesia and oncological radical resection with central lymphadenectomy. Exclusion criteria included recurrent colon cancer, ongoing treatment for other cancer and metastasis outside resection area. All patients gave written, informed consent to inclusion in the study, and a separate consent to collect and analyse biological tissue. Four patients were included in the Neo-Col protocol (Clinicaltrials.gov identifier NCT01108107) and three of them randomized to receive neo-ACT.

*Cell free DNA purification for AVENIO NGS-panel*

3-5 mL purified plasma (n = 62) was thawed prior to enrichment of cell free DNA by the AVENIO ctDNA Analysis Kit (Roche) according to the protocol provided by the producers. Briefly, 500 µl Proteinase K was added before incubation at room temperature (RT) for 5 minutes. Each sample was incubated with 4 mL DNA PBB for 30 minutes (RT) before adding 1000 µl Isopropanol and transferring the sample to the High Pure Extender Assembly Unit. The unit was centrifuged at 3250 x g for 5 min before the included Filter Tube was washed with 500 µl Wash Buffer I. The Filter Tube was centrifuged at 8000 x g for 1 minute and added 500 µl Wash Buffer II before new centrifugation (1 minute at 8000 x g, 1 minute at 16000 x g) and final elution in 65 µl DNA Elution Buffer (5 minutes incubation at RT, 1 minute at 8000 x g). The top 60 µl was transferred to a new tube before quantity analysis using the Qubit 2.0 Fluorometer (ThermoFisher), and quality analysis by the Agilent 2100 Bioanalyzer (Agilent), as recommended by the producers.

*Avenio ctDNA Surveillance Panel Library preparation*

Sequencing libraries were prepared from 13-50 ng cell free DNA using the AVENIO ctDNA Analysis Kit paired with the AVENIO ctDNA Surveillance Kit (both from Roche) as described by the manufacturers. After library enrichment, 11 labelled DNA samples were pooled, diluted to 2.25 nM, denatured and added PhiX Control V3 (Illumina) to a final concentration of 15-20%. The library was sequenced on a NextSeq550 (Illumina) using the NextSeq 500/550 High Output v2 kit (300 cycles) (Illumina).

*Tumour biopsy DNA purification for AVENIO Surveillance tissue kit*

DNA was purified using the QIAamp DNA Mini and Blood Mini kit (Qiagen) as recommended by the manufacturers. Briefly, 10-25 mg tissue was added 180 µl Buffer ATL and 20 µl Proteinase K, and lysed at 56$^{\circ}$C overnight. The solution was ethanol washed, and eluted from QIAamp Mini spin columns using 100 µl Buffer AE (10 mM Tris, 0.5 mM EDTA, pH 9.0), and stored as one aliquot at -80°C until analysis. DNA quantity and quality were assessed by the Qubit 2.0 Fluorometer (Invitrogen) and Agilent 2100 Bioanalyzer (Agilent), respectively.

*Avenio Tumour Tissue Surveillance Panel Library preparation*

Sequencing libraries were prepared from 100 µl DNA harvested from fresh frozen primary biopsies, as described above, using the AVENIO Tumour Tissue Analysis Kit paired with the AVENIO Tumour Surveillance Kit (both from Roche) as recommended by the manufacturers, with minor alterations. Briefly, a buffer exchange was done during the post-extraction cleanup using the kits Cleanup beads, before quality analysis on a LightCycler 480 Instrument II (Roche) and quantity analysis by the Qubit 2.0 Fluorometer. The input mass for each sample was calculated as follows: Q score = 2^(average(Cp66 bp amplicon)-average(Cp191 bp amplicon))^. Q-ratio = sample Q score/QC PCR DNA Standard Q score. Input mass in ng = 10/(Q-ratio)+10. DNA input ranged from 20 to 24 ng. After library preparation as recommended by the manufacturers, 24 labelled DNA samples were pooled, diluted to 2.25 nM and denatured. The library was sequenced on a NextSeq550 using the NextSeq 500/550 High Output v2 kit (300 cycles).

*Bioinformatics*

Results were analysed using AVENIO ctDNA Analysis Software version 2.0.0 (Roche) as recommended by the suppliers, with hg38 as reference genome. In short, BCL files from the Illumina NextSeq sequencer were accessed via the software and uploaded to the AVENIO server (Roche) where the analysis was performed. The default filter chain for the AVENIO Surveillance gene panel was chosen. The filter chain kept variants found in COSMIC v83, TCGA 9.0 or the Loci of Interest list, and removed variants found in ExAc 1.0 or 1000 Genomes (phase_3_v5b) having a MAF higher than 0.1%, in addition to removing variants fond in dbSNP Common v150. BAM files were investigated using Integrative Genomic Viewer (Broad Institute) for all filtered variants reported, and variants with strand bias, defined as one read direction representing more than 75% of the reads, were removed.

*Digital droplet PCR*

Cell free DNA was harvested from 4-5 mL purified plasma (n = 311) using the QIAamp Circulating Nucleic Acid Kit (Qiagen) according to the protocol provided by the producers. cfDNA was eluted in 55 µl Buffer AVE before quantity and quality assessment by the Qubit 2.0 Fluorometer (Invitrogen) and Agilent 2100 Bioanalyzer (Agilent), respectively. ddPCR assays for mutations discovered by the AVENIO Surveillance gene panel were purchased from Bio-Rad (Supplementary Table S1). Mutations chosen for monitoring were early hits in the clonal evolution of cancer, and in cases with multiple relevant mutations, the variant with highest VAF was chosen. ddPCR was performed as previously described (21), with minor alterations. Briefly, all samples were run as triplicates, and results are presented as average between replicates calculating number of mutant copies per mL plasma (or fractional abundance as mutant DNA copies/total DNA copies). All runs included positive controls (biopsy DNA), negative controls (cfDNA from healthy blood donors) and non-template controls for each assay. PCR program was as follows: 95°C for 10 minutes, 40 cycles of 94°C for 30 seconds and 55°C for 1 minute, 98°C for 10 minutes, 4°C hold. The last step was performed at 1°C/second, the remaining steps at 2°C/second. Results were analysed using the Quantasoft version 1.7.4 software (Bio-Rad), manually gating each assay based on positive controls, negative controls and non-template controls. Results were presented as % fractional abundance (FA). Samples with < 12,000 droplets generated per parallel were excluded from further analysis. Samples generating a total of < 3 mutation-positive droplets or having a FA < 0.1% were defined as having no detectable tumour DNA. The dMIQE2020 checklist for authors on ddPCR experiments is provided in Supplementary Table S3.

*Microsatellite instability (MSI) analysis*

MSI-status was evaluated by the MSI Analysis System, version 1.2 (Promega) using the ABI PRISM 3100 Genetic Analyzer (Thermo Fisher) as described by the producers. Briefly, the system evaluated seven markers; five mononucleotide loci and two pentanucleotide loci. Department of Pathology performed the MSI analysis routinely for 16 patients, whereas the remaining 32 patients were analysed by the research laboratory.

*Statistical analysis*

RFS was measured from the date of the fist postoperative sampling to the verified first radiologic recurrence (distant or local) or death from colon cancer recurrence, and was censored at last follow-up or non-colon cancer-related death. Potential predictors of recurrence were ctDNA positive preoperative, ctDNA positive postoperative, tumour stage (pT1-3 versus pT4), node stage (pN0 versus pN1-3), tumour differentiation (well/middle versus poor), morphology of tumour (adenocarcinoma versus signet ring cell carcinoma), mucinous differentiation, tumour deposit, venous invasion and MSI-status.

**Supplementary Figures and Tables**

**Supplementary Table S1.** Assay information for gene mutations monitored by ddPCR in 47 patients with right-sided colon cancer.

| Gene | c. Position | p. Position | Assay Information | Number of patient | Location of Amplicon (hg19) | Length of Amplicon | MIQE Context |
| --- | --- | --- | --- | --- | --- | --- | --- |
| APC | c.2626C>T | p.R876* | dHsaMDS444452080 | 3 | chr5:112173856-112173978 | 63 | ACGCGGAATTGGTCTAGGCAACTACCATCCAGCAACAGAAAATCCAGGAACTTCTTCAAAG[C/T]GAGGTTTGCAGATCTCCACCACTGCAGCCCAGATTGCCAAAGTCATGGAAGAAGTGTCAGC |
| APC | c.3955delC | p.P1319fs | dHsaMDS311136281 | 1 | chr5:112175185-112175307 | 65 | TGCTAATACCCTGCAAATAGCAGAAATAAAAGAAAAGATTGGAACTAGGTCAGCTGAAGAT[C/]CTGTGAGCGAAGTTCCAGCAGTGTCACAGCACCCTAGAACCAAATCCAGCAGACTGCAGGG |
| APC | c.3964G>T | p.E1322* | dHsaMDS2514046 | 1 | chr5:112175194-112175316 | 69 | CCTGCAAATAGCAGAAATAAAAGAAAAGATTGGAACTAGGTCAGCTGAAGATCCTGTGAGC[G/T]AAGTTCCAGCAGTGTCACAGCACCCTAGAACCAAATCCAGCAGACTGCAGGGTTCTAGTTT |
| APC | c.4222G>T | p.E1408* | dHsaMDS2512796 | 2 | chr5:112175452-112175574 | 65 | TACTTCTGTCAGTTCACTTGATAGTTTTGAGAGTCGTTCGATTGCCAGCTCCGTTCAGAGT[G/T]AACCATGCAGTGGAATGGTAAGTGGCATTATAAGCCCCAGTGATCTTCCAGATAGCCCTGG |
| APC | c.4348C>T | p.R1450* | dHsaMDV2510508 | 7 | chr5:112175578-112175700 | 63 | AACCATGCCACCAAGCAGAAGTAAAACACCTCCACCACCTCCTCAAACAGCTCAAACCAAG[C/T]GAGAAGTACCTAAAAATAAAGCACCTACTGCTGAAAAGAGAGAGAGTGGACCTAAGCAAGC |
| BRAF | c.1799T>A | p.V600E | dHsaMDV2010027 | 11 | chr7:140453075-140453197 | 91 | TCAGATATATTTCTTCATGAAGACCTCACAGTAAAAATAGGTGATTTTGGTCTAGCTACAG[T/A]GAAATCTCGATGGAGTGGGTCCCATCAGTTTGAACAGTTGTCTGGATCCATTTTGTGGATG |
| KRAS | c.34G>T | p.G12C | dHsaMDV2510584 | 1 | chr12:25398224-25398346 | 57 | ATTATTTTTATTATAAGGCCTGCTGAAAATGACTGAATATAAACTTGTGGTAGTTGGAGCT[G/T]GTGGCGTAGGCAAGAGTGCCTTGACGATACAGCTAATTCAGAATCATTTTGTGGACGAATA |
| KRAS | c.35G>A | p.G12D | dHsaMDV2510596 | 6 | chr12:25398223-25398345 | 57 | TTATTTTTATTATAAGGCCTGCTGAAAATGACTGAATATAAACTTGTGGTAGTTGGAGCTG[G/A]TGGCGTAGGCAAGAGTGCCTTGACGATACAGCTAATTCAGAATCATTTTGTGGACGAATAT |
| KRAS | c.34G>A | p.G12S | dHsaMDV2510588 | 1 | chr12:25398224-25398346 | 57 | ATTATTTTTATTATAAGGCCTGCTGAAAATGACTGAATATAAACTTGTGGTAGTTGGAGCT[G/A]GTGGCGTAGGCAAGAGTGCCTTGACGATACAGCTAATTCAGAATCATTTTGTGGACGAATA |
| KRAS | c.35G>T | p.G12V | dHsaMDV2510592 | 3 | chr12:25398223-25398345 | 57 | TTATTTTTATTATAAGGCCTGCTGAAAATGACTGAATATAAACTTGTGGTAGTTGGAGCTG[G/T]TGGCGTAGGCAAGAGTGCCTTGACGATACAGCTAATTCAGAATCATTTTGTGGACGAATAT |
| KRAS | c.38G>A | p.G13D | dHsaMDV2510598 | 3 | chr12:25398220-25398342 | 57 | TTTTTATTATAAGGCCTGCTGAAAATGACTGAATATAAACTTGTGGTAGTTGGAGCTGGTG[G/A]CGTAGGCAAGAGTGCCTTGACGATACAGCTAATTCAGAATCATTTTGTGGACGAATATGAT |
| KRAS | c.183A>C | p.Q61H | dHsaMDV2010133 | 2 | chr12:25380214-25380336 | 61 | GGAAGCAAGTAGTAATTGATGGAGAAACCTGTCTCTTGGATATTCTCGACACAGCAGGTCA[A/C]GAGGAGTACAGTGCAATGAGGGACCAGTACATGAGGACTGGGGAGGGCTTTCTTTGTGTAT |
| KRAS | c.436G>A | p.A146T | dHsaMDV2010079 | 3 | chr12:25378501-25378623 | 80 | AGACACAAAACAGGCTCAGGACTTAGCAAGAAGTTATGGAATTCCTTTTATTGAAACATCA[G/A]CAAAGACAAGACAGGTAAGTAACACTGAAATAAATACAGATCTGTTTTCTGCAAAATCATA |
| NRAS | c.181C>A | p.Q61K | dHsaMDV2010067 | 1 | chr1:115256469-115256591 | 65 | CAGAAAACAAGTGGTTATAGATGGTGAAACCTGTTTGTTGGACATACTGGATACAGCTGGA[C/A]AAGAAGAGTACAGTGCCATGAGAGACCAATACATGAGGACAGGCGAAGGCTTCCTCTGTGT |
| TP53 | c.524G>A | p.R175H | dHsaMDV2010105 | 4 | chr17:7578345-7578467 | 65 | ACCCGCGTCCGCGCCATGGCCATCTACAAGCAGTCACAGCACATGACGGAGGTTGTGAGGC[G/A]CTGCCCCCACCATGAGCGCTGCTCAGATAGCGATGGTGAGCAGCTGGGGCTGGAGAGACGA |
| TP53 | c.578A>G | p.H193R | dHsaMDV2516924 | 1 | chr17:7578210-7578332 | 65 | CCCAGGGTCCCCAGGCCTCTGATTCCTCACTGATTGCTCTTAGGTCTGGCCCCTCCTCAGC[A/G]TCTTATCCGAGTGGAAGGAAATTTGCGTGTGGAGTATTTGGATGACAGAAACACTTTTCGA |
| TP53 | c.734G>A | p.R248Q | dHsaMDV2010127 | 2 | chr17:7577477-7577599 | 62 | GACTGTACCACCATCCACTACAACTACATGTGTAACAGTTCCTGCATGGGCGGCATGAACC[G/A]GAGGCCCATCCTCACCATCATCACACTGGAAGACTCCAGGTCAGGAGCCACTTGCCACCCT |
| TP53 | c.817C>T | p.R273C | dHsaMDV2510538 | 5 | chr17:7577060-7577182 | 65 | TTGCTTCTCTTTTCCTATCCTGAGTAGTGGTAATCTACTGGGACGGAACAGCTTTGAGGTG[C/T]GTGTTTGTGCCTGTCCTGGGAGAGACCGGCGCACAGAGGAAGAGAATCTCCGCAAGAAAGG |
| TP53 | c.844C>T | p.R282W | dHsaMDV2516902 | 4 | chr17:7577033-7577155 | 64 | TGGTAATCTACTGGGACGGAACAGCTTTGAGGTGCGTGTTTGTGCCTGTCCTGGGAGAGAC[C/T]GGCGCACAGAGGAAGAGAATCTCCGCAAGAAAGGGGAGCCTCACCACGAGCTGCCCCCAGG |
| TP53 | c.1024C>T | p.R342* | dHsaMDV2516914 | 3 | chr17:7573942-7574064 | 63 | ACTTACTTCTCCCCCTCCTCTGTTGCTGCAGATCCGTGGGCGTGAGCGCTTCGAGATGTTC[C/T]GAGAGCTGAATGAGGCCTTGGAACTCAAGGATGCCCAGGCTGGGAAGGAGCCAGGGGGGAG |

**Supplementary Table S2.** Gene mutations detected by NGS in tumour and/or plasma followed by monitoring by ddPCR

| Patient | Biomarker | ctDNA analyzed by ddPCR? | VAF plasma (%) pre-op, NGS | VAF tissue (%), NGS | Tumor positive by ddPCR | Plasma positive by ddPCR# | Plasma pre-op positive by ddPCR | Assay ID |
| --- | --- | --- | --- | --- | --- | --- | --- | --- |
| 162 | BRAF p.V600E c.1799T>A | Yes | 0,16 | 27,2 | Yes | Yes | Yes | dHsaMDV2010027 |
| 162 | TP53 p.R175H c.524G>A | Yes | ND | 28,47 | Yes | No | NT | dHsaMDV2010105 |
| 164 | KRAS p.G12S c.34G>A | Yes | 0,71 | 49,71 | Yes | Yes | Yes | dHsaMDV2510588 |
| 164 | TP53 p.R248Q c.734G>A | Yes | * | 54,35 | Yes | No | NT | dHsaMDV2010127 |
| 165 | KRAS p.A146T c.436G>A | Yes | 0,17 | 17,81 | Yes | Yes | No | dHsaMDV2010079 |
| 165 | TP53 p.V173L c.517G>T | No | 0,3 | 21,28 | - | - | - | - |
| 168 | BRAF p.V600E c.1799T>A | Yes | 0,26 | 17,12 | Yes | Yes | NT | dHsaMDV2010027 |
| 170 | KRAS p.G12D c.35G>A | Yes | 0,18 | 23,64 | Yes | No | No | dHsaMDV2510596 |
| 172 | APC p.E1408* c.4222G>T | Yes | NT | 28,79 | Yes | No | No | dHsaMDS2512796 |
| 172 | KRAS p.G12D c.35G>A | Yes | NT | 36,72 | Yes | No | No | dHsaMDV2510596 |
| 176 | BRAF p.V600E c.1799T>A | Yes | 0,03 | 20,05 | Yes | Yes | No | dHsaMDV2010027 |
| 176 | TP53 p.R282W c.844C>T | Yes | * | 32,75 | Yes | No | No | dHsaMDV2516902 |
| 178 | BRAF p.V600E c.1799T>A | Yes | 0,48 | 27,26 | Yes | Yes | Yes | dHsaMDV2010027 |
| 179 | APC p.R876* c.2626C>T | Yes | 0,10 | 29,53 | Yes | No | NT | dHsaMDS444452080 |
| 179 | KRAS p.G12D c.35G>A | Yes | * | 44,69 | Yes | No | No | dHsaMDV2510596 |
| 181 | BRAF p.V600E c.1799T>A | Yes | 0,06 | 5,66 | Yes | Yes | No | dHsaMDV2010027 |
| 182 | No variant detected | - | - | - | - | - | - | - |
| 187 | KRAS p.G12V c.35G>T | Yes | * | 19,72 | Yes | No | No | dHsaMDV2510592 |
| 187 | PIK3CA p.E545K c.1633G>A | No | * | 18,90 | - | - | - | - |
| 190 | KRAS p.Q61H c.183A>C | Yes | * | 32,40 | Yes | No | No | dHsaMDV2510598 |
| 191 | BRAF p.V600E c.1799T>A | Yes | 0,27 | 14,10 | Yes | Yes | No | dHsaMDV2010027 |
| 191 | TP53 p.R273C c.817C>T | Yes | 0,32 | 36,09 | Yes | Yes | Yes | dHsaMDV2510538 |
| 193 | KRAS p.A146T c.436G>A | Yes | 1,65 | 35,00 | Yes | Yes | Yes | dHsaMDV2010079 |
| 193 | TP53 p.R273C c.817C>T | Yes | 0,95 | 31,17 | Yes | Yes | Yes | dHsaMDV2510538 |
| 194 | BRAF p.V600E c.1799T>A | Yes | 0,60 | 19,80 | Yes | Yes | Yes | dHsaMDV2010027 |
| 195 | APC p.R876* c.2626C>T | Yes | ND | 36,55 | Yes | No | NT | dHsaMDS444452080 |
| 195 | TP53 p.R273C c.817C>T | Yes | 0,89 | 0,58 | Yes | Yes | Yes | dHsaMDV2510538 |
| 196 | KRAS p.G12D c.35G>A | Yes | ND | 20,06 | Yes | No | No | dHsaMDV2510596 |
| 197 | APC p.E1322* c.3964G>T | Yes | 0,61 | NT | Yes | Yes | Yes | dHsaMDS2514046 |
| 197 | KRAS p.G13D c.38G>A | Yes | 0,11 | NT | Yes | Yes | No | dHsaMDV2510598 |
| 198 | TP53 p.R273C c.817C>T | Yes | 0,43 | 46,19 | Yes | Yes | Yes | dHsaMDV2510538 |
| 201 | APC p.R1450* c.4348C>T | Yes | ND | 20,60 | Yes | No | No | dHsaMDV2510508 |
| 201 | KRAS p.Q61H c.183A>C | Yes | 0,07 | 24,49 | Yes | No | No | dHsaMDV2510598 |
| 202 | BRAF p.V600E c.1799T>A | Yes | 2,08 | NT | Yes | Yes | Yes | dHsaMDV2010027 |
| 215 | BRAF p.V600E c.1799T>A | Yes | 0,48 | 4,40 | Yes | No | No | dHsaMDV2010027 |
| 215 | TP53 p.R175H c.524G>A | Yes | * | 6,20 | Yes | Yes | No | dHsaMDV2010105 |
| 216 | APC p.P1319fs c.3956delC/c.3955delC (same variant) | Yes | 2,16 | NT | Yes | Yes | Yes | dHsaMDV2510508 |
| 218 | BRAF p.V600E c.1799T>A | Yes | 1,92 | 27,02 | Yes | Yes | Yes | dHsaMDV2010027 |
| 219 | APC p.R876* c.2626C>T | Yes | ND | 24,76 | Yes | No | No | dHsaMDS444452080 |
| 219 | KRAS p.G12C c.34G>T | Yes | * | 37,71 | Yes | No | No | dHsaMDV2510584 |
| 220 | BRAF p.V600E c.1799T>A | Yes | 0,26 | 18,67 | Yes | Yes | Yes | dHsaMDV2010027 |
| 221 | KRAS p.G13D c.38G>A | Yes | 1,58 | ND | Yes | Yes | Yes | dHsaMDV2510598 |
| 221 | TP53 p.R175H c.524G>A | Yes | 2,29 | ND | Yes | Yes | Yes | dHsaMDV2010105 |
| 222 | APC p.R1450* c.4348C>T | Yes | NT | 6,69 | Yes | No | No | dHsaMDV2510508 |
| 222 | BRAF p.V600E c.1799T>A | Yes | NT | 11,88 | Yes | No | No | dHsaMDV2010027 |
| 223 | KRAS p.G12V c.35G>T | Yes | NT | 16,35 | Yes | Yes | Yes | dHsaMDV2510592 |
| 223 | TP53 p.R273C c.817C>T | Yes | NT | 24,41 | Yes | No | No | dHsaMDV2510538 |
| 225 | APC p.R1450* c.4348C>T | Yes | NT | 20,08 | Yes | Yes | Yes | dHsaMDV2510508 |
| 225 | NRAS p.Q61K c.181C>A | Yes | NT | 20,29 | Yes | Yes | Yes | dHsaMDV2010067 |
| 228 | TP53 p.R342* c.1024C>T | Yes | NT | 64,60 | Yes | No | No | dHsaMDV2516914 |
| 232 | BRAF p.V600E c.1799T>A | Yes | NT | 22,00 | Yes | No | No | dHsaMDV2010027 |
| 232 | TP53 p.R282W c.844C>T | Yes | NT | 21,95 | Yes | No | No | dHsaMDV2516902 |
| 235 | APC p.R1450* c.4348C>T | Yes | NT | 80,47 | Yes | Yes | No | dHsaMDV2510508 |
| 235 | KRAS p.A146T c.436G>A | Yes | NT | 80,58 | Yes | No | No | dHsaMDV2010079 |
| 236 | APC p.R1450* c.4348C>T | Yes | NT | 32,96 | Yes | No | No | dHsaMDV2510508 |
| 236 | BRAF p.V600E c.1799T>A | Yes | NT | 24,16 | Yes | No | No | dHsaMDV2010027 |
| 237 | No relevant variant | - | - | - | - | - | - | - |
| 239 | APC p.E1408* c.4222G>T | Yes | NT | 37,39 | Yes | Yes | Yes | dHsaMDS2512796 |
| 239 | KRAS p.G12F c.34_35GG>TT | No | NT | 52,87 | - | - | - | - |
| 240 | APC p.R1450* c.4348C>T | Yes | NT | 47,09 | Yes | No | No | dHsaMDV2510508 |
| 240 | KRAS p.G12A c.35G>C | No | NT | 31,43 | - | - | - | - |
| 241 | KRAS p.G12D c.35G>A | Yes | NT | 21,52 | Yes | No | No | dHsaMDV2510596 |
| 241 | TP53 p.R248Q c.734G>A | Yes | NT | 28,55 | Yes | No | No | dHsaMDV2010127 |
| 244 | BRAF p.V600E c.1799T>A | Yes | NT | 29,13 | Yes | Yes | Yes | dHsaMDV2010027 |
| 244 | TP53 p.R282W c.844C>T | Yes | NT | 29,74 | Yes | Yes | Yes | dHsaMDV2516902 |
| 257 | BRAF p.V600E c.1799T>A | Yes | NT | 33,91 | Yes | Yes | Yes | dHsaMDV2010027 |
| 258 | BRAF p.V600E c.1799T>A | Yes | NT | 45,36 | Yes | No | No | dHsaMDV2010027 |
| 258 | TP53 p.G245S c.733G>A | No | NT | 82,86 | - | - | - | - |
| 260 | KRAS p.G12V c.35G>T | Yes | NT | 22,72 | Yes | Yes | Yes | dHsaMDV2510592 |
| 260 | TP53 p.G266E c.797G>A | Yes | NT | 37,73 | Yes | Yes | Yes | dHsaMDV2516854 |
| 261 | KRAS p.G12A c.35G>C | No | NT | 40,73 | - | - | - | - |
| 261 | TP53 p.R175H c.524G>A | Yes | NT | 76,88 | Yes | No | No | dHsaMDV2010105 |
| 265 | KRAS p.G12D c.35G>A | Yes | NT | 3,35 | Yes | No | No | dHsaMDV2510596 |
| 265 | KRAS p.G13D c.38G>A | Yes | NT | 32,83 | Yes | No | No | dHsaMDV2510598 |
| 267 | APC p.R1450* c.4348C>T | Yes | 0,17 | NT | Yes | Yes | Yes | dHsaMDV2510508 |
| 268 | BRAF p.V600E c.1799T>A | Yes | NT | 17,76 | Yes | Yes | Yes | dHsaMDV2010027 |
| 268 | TP53 p.R342* c.1024C>T | Yes | NT | 19,61 | Yes | Yes | Yes | dHsaMDV2516914 |
| 269 | TP53 p.H193R c.578A>G | Yes | NT | 38,42 | Yes | Yes | Yes | dHsaMDV2516924 |
| 270 | BRAF p.V600E c.1799T>A | Yes | NT | 12,98 | Yes | No | No | dHsaMDV2010027 |
| 270 | TP53 p.R282W c.844C>T | Yes | NT | 23,59 | Yes | Yes | Yes | dHsaMDV2516902 |
| 291 | No relevant variant | - | - | NT | - | - | - | - |
|  |  |  |  |  |  |  |  |  |
| VAF = variant allele fraction | |  |  |  |  |  |  |  |
|  |  |  |  |  |  |  |  |  |
| # in any sample tested | |  |  |  |  |  |  |  |
| * detected in raw data (BAM file) | |  |  |  |  |  |  |  |
| NT - not tested | |  |  |  |  |  |  |  |
| ND - not detected | |  |  |  |  |  |  |  |

**Supplementary Table S3.** MIQE (Minimum Information for table for Publication of Quantitative Digital PCR Experiments for 2020) table

|  | |  |
| --- | --- | --- |
|  |  |  |
|  |  |  |
| **ITEM TO CHECK** | **PROVIDED** | **COMMENT** |
| **Column1** | **Y/N** | **Column2** |
| **1. SPECIMEN** |  |  |
| Detailed description of specimen type and numbers | **Y** | Described in "*Digital droplet PCR"* and "*Tumor biopsy DNA purification for AVENIO Surveillance tissue kit"* in materials and methods |
| Sampling procedure (including time to storage) | **Y** | Described in "*Sample collection"* in materials and methods |
| Sample aliquotation, storage conditions and duration | **Y** | Described in "*Sample collection*" in materials and methods |
| **2. NUCLEIC ACID EXTRACTION** |  |  |
| Description of extraction method including amount of sample processed | **Y** | Described in "*Tumor biopsy DNA purification for AVENIO Surveillance tissue kit*" and "*Digital droplet PCR*" in materials and methods |
| Volume of solvent used to elute/resuspend extract | **Y** | Described in "Tumor biopsy DNA purification for AVENIO Surveillance tissue kit" and "Digital droplet PCR" in materials and methods |
| Number of extraction replicates | **Y** | Extraction replicates were performed for only 4 of the samples due to limited sample material |
| Extraction blanks included? | **Y** | Extraction blanks were not included |
| **3. NUCLEIC ACID ASSESSMENT AND STORAGE** |  |  |
| Method to evaluate quality of nucleic acids | **Y** | Described in "Tumor biopsy DNA purification for AVENIO Surveillance tissue kit" and "Digital droplet PCR" in materials and methods |
| Method to evaluate quantity of nucleic acids (including molecular weight and calculations when using mass) | **Y** | Described in "Tumor biopsy DNA purification for AVENIO Surveillance tissue kit" and "Digital droplet PCR" in materials and methods |
| Storage conditions: temperature, concentration, duration, buffer, aliquots | **Y** | Described in "Tumor biopsy DNA purification for AVENIO Surveillance tissue kit" and "Digital droplet PCR" in materials and methods |
| Clear description of dilution steps used to prepare working DNA solution | **Y** | Only positive control DNA was diluted. This was done by adding dd H_2_O |
| **4. NUCLEIC ACID MODIFICATION** | **N** | No template modification was performed |
| Template modification (digestion, sonication, pre-amplification, bisulphite etc.) | **N** | Not applicable |
| Details of repurification following modification if performed | **N** | Not applicable |
| **5. REVERSE TRANSCRIPTION** | **N** | No reverse transcription was performed |
| cDNA priming method and concentration | **N** | Not applicable |
| One or two step protocol (include reaction details for two step) | **N** | Not applicable |
| Amount of RNA added per reaction | **N** | Not applicable |
| Detailed reaction components and conditions | **N** | Not applicable |
| Estimated copies measured with and without addition of RT* | **N** | Not applicable |
| Manufacturer of reagents used with catalogue and lot numbers | **N** | Not applicable |
| Storage of cDNA: temperature, concentration, duration, buffer and aliquots | **N** | Not applicable |
| **6. dPCR OLIGONUCLEOTIDES DESIGN AND TARGET INFORMATION** |  |  |
| Sequence accession number or official gene symbol | **Y** | Provided in Table 2 |
| Method (software) used for design and *in silico* verification | **Y** | All primers and probes were pre-designed by Bio-Rad |
| Location of amplicon | **Y** | Provided in Supplementary Table 1 |
| Amplicon length | **Y** | Provided in Supplementary Table 1 |
| Primer and probe sequences (or amplicon context sequence)** | **Y** | Provided in Supplementary Table 1 |
| Location and identity of any modifications | **Y** | No modifications were made |
| Manufacturer of oligonucleotides | **Y** | Bio-Rad |
| **7. dPCR PROTOCOL** |  |  |
| Manufacturer of dPCR instrument and instrument model | **Y** | Bio-Rad, QX200 AutoDG Droplet Digital PCR System, Quantasoft version 1.7.4 software |
| Buffer/kit manufacturer with catalogue and lot number | **Y** | Bio-Rad + Provided in Supplementary Table 1 |
| Primer and probe concentration | **Y** | Described in "*Digital droplet PCR*" in materials and methods |
| Pre-reaction volume and composition (incl. amount of template and if restriction enzyme added) | **Y** | Described in "*Digital droplet PCR*" in materials and methods |
| Template treatment (initial heating or chemical denaturation) | **Y** | No sample pre-treatment was performed |
| Polymerase identity and concentration, Mg++ and dNTP concentrations*** | **N** | As described by the producers (Bio-Rad) |
| Complete thermocycling parameters | **Y** | Described in "*Digital droplet PCR*" in materials and methods |
| **8. ASSAY VALIDATION** |  |  |
| Details of optimisation performed | **N** | All assays were optimized by the producer |
| Analytical specificity (vs. related sequences) and limit of blank (LOB) | **Y** | LoB was no mutant positive droplets |
| Analytical sensitivity/LoD and how this was evaluated | **Y** | 3 positive droplets per triplicate is LoD per assay |
| Testing for inhibitors (from biological matrix/extraction) | **N** | Inhibitors were not tested |
| **9. DATA ANALYSIS** |  |  |
| Description of dPCR experimental design | **Y** | Described in "*Digital droplet PCR*" in materials and methods |
| Comprehensive details negative and positive of controls (whether applied for QC or for estimation of error) | **Y** | Described in "*Digital droplet PCR*" in materials and methods |
| Partition classification method (thresholding) | **Y** | Described in "*Digital droplet PCR*" in materials and methods |
| Examples of positive and negative experimental results (including fluorescence plots in supplemental material) | **Y** | Described in Supplementary Information |
| Description of technical replication | **Y** | All samples were run in triplicates |
| Repeatability (intra-experiment variation) | **Y** | All samples were run in triplicates |
| Reproducibility (inter-experiment/user/lab etc. variation ) | **Y** | All samples were run in triplicates |
| Number of partitions measured (average and standard deviation ) | **Y** | Minimum droplets for valid experiments were 12 000 per well |
| Partition volume | **Y** | 0,85 nl |
| Copies per partition (λ or equivalent ) (average and standard deviation) | **N** | Not known |
| dPCR analysis program (source, version) | **Y** | Quantasoft version 1.7.4 software |
| Description of normalisation method | **N** | Normalisation not performed |
| Statistical methods used for analysis | **Y** | See section for statistics in Material and Methods |
| Data transparency |  | Raw data can be provided upon request |
|  |  |  |
|  |  |  |
|  |  |  |
| * Assessing the absence of DNA using a no RT assay (or where RT has been inactivated) is essential when first extracting RNA. Once the sample has been validated as DNA-free, inclusion of a no-RT control is desirable, but no longer essential. |  |  |
|  |  |  |
| ** Disclosure of the primer and probe sequence is highly desirable and strongly encouraged. However, since not all commercial pre-designed assay vendors provide this information when it is not available assay context sequences must be submitted (Bustin et al. Primer sequence disclosure: A clarification of the miqe guidelines. Clin Chem 2011;57:919-21.) |  |  |
|  |  |  |
| *** Details of reaction components is highly desirable, however not always possible for commercial disclosure reasons. Inclusion of catalogue number is essential where component reagent details are not available. |  |  |

**Supplementary figure 1 Remark checklist**

| **Item to be reported** | | **Page no.** |
| --- | --- | --- |
| **INTRODUCTION** | |  |
| 1 | State the marker examined, the study objectives, and any pre-specified hypotheses. | 4 |
| **MATERIALS AND METHODS** | |  |
| *Patients* | |  |
| 2 | Describe the characteristics (e.g., disease stage or co-morbidities) of the study patients, including their source and inclusion and exclusion criteria. | 4, S1 |
| 3 | Describe treatments received and how chosen (e.g., randomized or rule-based). | 5 |
| *Specimen characteristics* | |  |
| 4 | Describe type of biological material used (including control samples) and methods of preservation and storage. | 5, 6, S1, S2, S3 |
| *Assay methods* | |  |
| 5 | Specify the assay method used and provide (or reference) a detailed protocol, including specific reagents or kits used, quality control procedures, reproducibility assessments, quantitation methods, and scoring and reporting protocols. Specify whether and how assays were performed blinded to the study endpoint. | 5, 6, S1, S2, S3 |
| *Study design* | |  |
| 6 | State the method of case selection, including whether prospective or retrospective and whether stratification or matching (e.g., by stage of disease or age) was used. Specify the time period from which cases were taken, the end of the follow-up period, and the median follow-up time. | 4 |
| 7 | Precisely define all clinical endpoints examined. | 7 |
| 8 | List all candidate variables initially examined or considered for inclusion in models. | S4 |
| 9 | Give rationale for sample size; if the study was designed to detect a specified effect size, give the target power and effect size. | 4 |
| *Statistical analysis methods* | |  |
| 10 | Specify all statistical methods, including details of any variable selection procedures and other model-building issues, how model assumptions were verified, and how missing data were handled. | 7, S4 |
| 11 | Clarify how marker values were handled in the analyses; if relevant, describe methods used for cutpoint determination. | 5,6,7, S1, S2, S3, S4 |
| **RESULTS** | |  |
| *Data* | |  |
| 12 | Describe the flow of patients through the study, including the number of patients included in each stage of the analysis (a diagram may be helpful) and reasons for dropout. Specifically, both overall and for each subgroup extensively examined report the numbers of patients and the number of events. | T2 |
| 13 | Report distributions of basic demographic characteristics (at least age and sex), standard (disease-specific) prognostic variables, and tumor marker, including numbers of missing values. | T1 |
| *Analysis and presentation* | |  |
| 14 | Show the relation of the marker to standard prognostic variables. | T4 |
| 15 | Present univariable analyses showing the relation between the marker and outcome, with the estimated effect (e.g., hazard ratio and survival probability). Preferably provide similar analyses for all other variables being analyzed. For the effect of a tumor marker on a time-to-event outcome, a Kaplan-Meier plot is recommended. | T4, F1 |
| 16 | For key multivariable analyses, report estimated effects (e.g., hazard ratio) with confidence intervals for the marker and, at least for the final model, all other variables in the model. | T4 |
| 17 | Among reported results, provide estimated effects with confidence intervals from an analysis in which the marker and standard prognostic variables are included, regardless of their statistical significance. | T4 |
| 18 | If done, report results of further investigations, such as checking assumptions, sensitivity analyses, and internal validation. | S3 |
| **DISCUSSION** | |  |
| 19 | Interpret the results in the context of the pre-specified hypotheses and other relevant studies; include a discussion of limitations of the study. | 10,11,12,13 |
| 20 | Discuss implications for future research and clinical value. | 13 |
